# Anti-biofilm efficacy of nano calcium hydroxide and chitosan nanoparticles against*E. faecalis*

**DOI:** 10.6026/973206300221126

**Published:** 2026-02-28

**Authors:** Aakriti Bansal, Ashwini Gaikwad, Aniket Jadhav, Ruchira Bhamare, Rajlaxmi Patil

**Affiliations:** 1 Department of Conservative Dentistry and Endodontics, Bharati Vidyapeeth Deemed to be University Dental College and Hospital, Pune, India

**Keywords:** Antibacterial efficacy, biofilm, chitosan nanoparticles, *Enterococcus faecalis*, intracanal medicament, nano calcium hydroxide root canal disinfection

## Abstract

*Enterococcus faecalis* is among the microorganisms frequently isolated from root canal infections. The outcome of the endodontic treatment depends on the effective disinfection of the root canal system. Therefore, it is of interest to assess the potential of Nano Calcium Hydroxide and Chitosan Nanoparticles as intracanal medicaments against *Enterococcus faecalis* biofilm. Hence, a total of twenty-eight single-rooted dental elements were included, where the incisal end of individual elements was infected with *Enterococcus faecalis*. The two medicaments used as intracanal dressing materials were Nano Calcium Hydroxide and Chitosan Nanoparticles. *Enterococcus faecalis* bacteria exposed to Nano Calcium Hydroxide was significantly higher as compared to those exposed to Chitosan Nanoparticles.

## Background:

Successful endodontic therapy is the foundation of dental practice; however, the outcome of the treatment depends enormously on the effective disinfection of the root canal system. With all the advances in Rotary instrumentation, ultrasonic activation and improved irrigants, it remains impossible to completely sterilize the root canal system [1]. This inadequate disinfection results from the complex anatomy of the root canal system that comprises lateral canals, isthmuses, apical deltas and dentinal tubules, which provide niches for the existence of microorganisms beyond mechanical and chemical debridement [2]. Enterococcus facials are among the microorganisms frequently isolated from root canal infections, particularly from cases of chronic apical periodontitis [3, 4]. This persistence could be related to the bacterium's ability to penetrate dentinal tubules, survive under nutrient-deprived conditions and resist high pH, antibiotics and antiseptic agents [5, 6]. *E. facials* cope with its alkaline surroundings by using proton pump mechanisms that maintain intracellular homeostasis [5]. Resistance to antimicrobial agents is further enhanced by the mature biofilm formation process since the biofilm neutralizes the disinfectants before they actually reach the bacterial cells [7]. Calcium hydroxide [CA (OH) _2_] has traditionally been considered the gold standard intracanal medicament because of its high alkalinity (pH ≈ 12.5), which disrupts bacterial cell membranes, denatures proteins and inactivates essential enzymes [8]. However, this product's effectiveness is limited against resistant microorganisms such as E. Faecalis by dentin buffering and a protective biofilm matrix that limits hydroxyl ion diffusion and penetration into dentinal tubules [4, 9]. Nanotechnology represents an emerging promising approach in endodontic disinfection, with nanoparticles in the 1-100 nm range demonstrating unique physicochemical properties, including an increased surface area-to-volume ratio that increases microbial interaction [10]. Such properties make possible improved penetration into complex anatomical structures such as lateral canals and dentinal tubules, as well as sustained antimicrobial ion release capability [11]. In addition, surface modification of nanoparticles enhances adhesion to bacterial membranes, facilitating targeted antimicrobial activity at lower concentrations [10, 12]. Chitosan nanoparticles, derived from a naturally occurring biopolymer chitosan, possess a positive surface charge, which enables them to strongly attract negatively charged bacterial membranes, causing loses and death [13]. Chitosan nanoparticles have been found to be effective agents because they release medicament slowly, a phenomenon referred to as sustained release [14]. Though literature shows the antimicrobial activity of nano calcium hydroxide and chitosan nanoparticles, few studies have examined their relative efficiencies in removing fully formed E. Faecalis biofilms using a standardized model [11, 12 and 14]. The colony-forming unit (CFU) test is widely accepted as a valid end point for studying the antimicrobial activity of agents [15]. Therefore, it is of interest to assess the potential of Nano Calcium Hydroxide and Chitosan Nanoparticles as intracanal medicaments against Enterococcus faecalis biofilm.

## Materials and Methods:

A total of twenty-eight extracted human intact human premolars with one single root and one single canal were selected for the present study. Samples were collected anonymously from the Department of Oral and Maxillofacial Surgery, Bharati Vidyapeeth (Deemed to be University) Dental College and Hospital, Pune. Ethical clearance was taken from the Institutional Ethics Committee (Ref. No. BVDU/DCH/5/ 2023-24). The research also took informed consent from the patients for the extracted teeth. Following this, the teeth were thoroughly cleaned of soft tissue debris and calculus using periodontal scalers. They were then preserved in normal saline solution, with the solution being changed every week to prevent bacterial contamination. The sample size was calculated based on Epic Info software version 3.01 using 80% power calculation with 95% confidence intervals, which requires 28 samples consisting of seven samples per subgroup. All teeth were decoronated at or below cement-enamel junction to standardize root length to 15 mm with a diamond disc under a water coolant. Radiographs were obtained to confirm single canal anatomy. Following preparation of access cavities, working length was established using a #10 K-file to 14 mm. Cleaning and shaping used ProTaper Gold rotary files to F3 (300 rpm, 3.0 Ncm torque).Saline and sodium hypochlorite (5.25%) were used as irrigation agents between files. For smear layer removal, 17% EDTA was then followed by sodium hypochlorite (5.25%). Prepared samples were sterilized through autoclaving at 121 °C and 15 psi pressure for 20 minutes. Sterility of specimens was confirmed by means of incubating three samples selected at random with Brain Heart Infusion broth; lack of turbidity indicated successful sterilization. Enterococcus faecalis-ATCC 29212 was grown in BHI broth at 37 °C for 24 h and then adjusted to 0.5 McFarland standards. Sterile root canals were inoculated with bacterial suspension by using a sterile micropipette and placed for incubation at 37 °C to develop biofilm. Fresh BHI broth was replaced periodically to keep the bacteria viable. Biofilms were allowed to develop for 6 weeks and 10 weeks in order to simulate an intermediate and mature stage of infection, respectively. Obtained samples were then divided, by randomization, into two groups (n = 14) depending on the medication used: Group A - Nano calcium hydroxide and Group B - 2% Chitosan nanoparticles. Further, each group was divided into two subgroups (n = 7) based on age of biofilm: A1 and B1 (6 weeks biofilm) and A2 and B2 (10 weeks biofilm) (Table 1).

Nano calcium hydroxide was prepared with distilled water as a vehicle to a semi-solid paste and was placed into canals using a lentos spiral. A liquid 2% chitosan nanoparticle solution was delivered by using a sterile syringe. After medicament placement, the canals were sealed with a temporary restorative material and incubated at 37 °C for one week. After medicament removal by using distilled water and drying with paper points, dentin debris was retrieved by #4 and #5 Gates-Glidden drills. Dentin chips were suspended in sterile saline, vortexes and serially diluted up to 10^-6^. One millilitre aliquots were plated in triplicate using the pour plate method on Soybean Casein Digest Agar. Plates were incubated at 30-37 °C for 24-48 hours and colonies between 30-300 CFU were counted for analysis. Data was entered, tabulated and analyzed using Microsoft Excel (2017) and IBM SPSS version 26.0.All the study parameters were subjected to both descriptive and inferential statistical analyses. Data distribution was tested for normality by performing the Kolmogorov-Smirnov test. Intergroup comparisons were done using the independent t-test, while intragroup comparisons at 6 and 10 weeks were done using the paired t-test. Statistical significance was achieved at a p <0.05 level

## Results:

At 6 weeks (Table 2 and Table 3), chitosan nanoparticles showed greater *E. faecalis* colony counts compared to nano calcium hydroxide, although this difference was not statistically significant (p = 0.233). After 10 weeks, nano calcium hydroxide was found to be significantly superior to chitosan nanoparticles in reducing colony counts (p = 0.043). The intergroup reduction in colony count from 6 to 10 weeks was calculated and it was noted that both nano calcium hydroxide and chitosan nanoparticles showed a reduction in colony counts, although not significant (p = 0.288 and p = 0.137 respectively) (Table 4).

## Discussion:

Persistence of endodontic infection still remains a challenge in endodontic therapy despite advancements in canal debridement and shaping procedures. The persistence of infection is usually dependent on the ability of microorganisms to survive and persist in the anatomy of the canal system and to form biofilm [1, 2]. Amongst the endodontic microorganisms, Enterococcus faecalis has been most commonly linked with endodontic infection owing to its increased resistance to intracanal antimicrobial agents, dentinal tubule penetration, collagen adhesion and survival under conditions of low nutrient availability and highly alkaline pH in the presence of calcium hydroxide [4 , 5]. *E. Faecalis* has a high potential for biofilm formation, which increases antimicrobial resistance; this property closely resembles endodontic infection, hence making it the most appropriate microorganism for biofilm formation studies *in vitro* [6]. Biofilm formation is a critical factor in the recalcitrance of the root canal infections. An extracellular polymeric substance matrix covers the microorganisms, restricts the uptake of antimicrobials, enhances intercellular communication and allows their survival as dormant and resistant bacterial populations, which can lead to failure of endodontic treatments [2, 3]. Six-week-old and 10-week-old biofilms have been used in the present study to represent the intermediate and mature stages of *E. Faecalis* biofilm development. Aging is known to create significant changes in antimicrobial susceptibility and it always happens that older biofilms show increased levels of resistance [7]. Ten-week biofilms more correctly represent well-established clinical infections and thus contribute significantly to translational relevance [10]. The canals were standardized using ProTaper Gold rotary files because the geometry of the canal significantly affects bacterial adhesion, biofilm formation and medicament penetration. Single-rooted premolars are selected to reduce anatomical variability, therefore enhancing reproducibility but still with clinical relevance. Calcium hydroxide is commonly used as an intracanal medicament because of its high alkalinity and antimicrobial activity. The mechanism of action primarily involves the release of hydroxyl ions, which interfere with the bacterial cell membrane and enzymatic activity. Nevertheless, the effectiveness of calcium hydroxide is reduced when co-existing with the Enterococcus faecalis biofilm, especially in dentinal tubules [9]. This reduction is due to the dentin buffering action, bacterial proton pumps and the poor diffusion of the medication into the dentinal tubules. The size of the calcium hydroxide particles (1-10 µm) is larger than the tubule size (approximately 2-2.5 µm), thus inhibiting the entry of the medication and the bacteria into the dentinal tubules and entrapping them [10]. Moreover, the short-term use does not maintain the required lethal pH value because of the dentin buffering action [13]. Chitosan, a biopolymer derived from an anionic polysaccharide, is being considered due to its antimicrobial activity, biocompatibility and bio-adhesiveness [11]. The antimicrobial activity of chitosan is due to the electrostatic attraction between the positive charge on the amino groups and the negative charge on the bacterial cell membrane, causing the membranes to be more permeable and leading to cell death [11]. Chitosan has shown promise against *E. Faecalis* through the disruption of biofilm architecture and adhesion to dentin [12]. The effectiveness of chitosan against microbes is affected by its molecular weight, deactivation and pH, with reduced activity in the presence of the physiological conditions ranging from neutral to alkaline in the root canal [11]. To overcome the drawbacks related to conventional medicaments, nanoparticle-based formulations have been developed, such as nano calcium hydroxide (NCH) and chitosan nanoparticles (CSNP). NCH offers sustained release of hydroxyl ions, offering sustained lethal alkalinity and resisting dentinal buffering. CSNP allows better penetration and targets infected biofilms through electrostatic forces, thus improving the overall antimicrobial action of intracanal medicaments. Intragroup analysis (Table 4) revealed a reduction in CFU from 6 to 10 weeks in the NCH group, although this was not found to be statistically significant (p value = 0.288). This study confirms previously described findings suggesting that antimicrobial properties of calcium hydroxide vary depending on prolonged hydroxyl ions percolation and continuous elevation of pH levels [8]. Changes in antimicrobial activities related to chitosan nanoparticles could be ascribed to physicochemical properties like molecular weights and charges [11]. Intergroup comparison (Table 3) found no statistical significant differences between NCH and CSNP at 6 weeks; thus, both materials have equal efficiency against younger biofilms. However, at 10 weeks, NCH had significantly reduced CFU count compared to CSNP (p = 0.043). Hence, NCH has better antibacterial efficacy against biofilms. The findings of the study coincide with previous studies concerning the potency of nano-calcium hydroxide against well-established *E. Faecalis* biofilms [13,14- 15]. Another notable observation was the reduction in the counts of CFU at 10 weeks in all medicaments. These delayed effects most likely point to the substantively of the medicaments used. This effect has been reported in the past and this most likely explains the prolonged retention of medicaments within the dentin [15, 12]. Nasr *et al.* concluded that, nano chitosan intracanal medication was effective against E. Faecalis biofilm compared to Calcium hydroxide [16]. Similarly Pandey *et al.* stated that, antimicrobial effectiveness of CSNPs was greater compared to calcium hydroxide and normal saline against *E. faecalis* biofilm [17]. The reduced counts may be due to the slow biofilm degradation, buffering effect of the dentin and the killing of the persisted cells. The novelty of the present study is the consideration of the nanoparticle-based intracanal medicament in association with the maturity of the biofilms by the 6- and 10-week-old *E. Faecalis* biofilm cultures, closely mimicking the realistic clinical scenario. The results suggest a tier-based disinfection paradigm in which the initial use of CSNP is followed by the prolonged use of NCH. This mechanism-based, kinetics-driven context of treatment marks a paradigm shift from the so farused monomeric-based disinfection therapy. Furthermore, within the limitations of this in vitro study, the null hypothesis was partially rejected. A statistically significant difference was seen at 10 weeks but not at 6 weeks. NCH exhibited better sustained antibacterial activity against well-formed*E. Faecalis* biofilms, indicating potential clinical relevance of nano CAH in managing persisting endodontic infection. To completely eradicate intra-radicular infections, chemical and mechanical preparations must be carried out in endodontic procedure. It may be possible to use nanoparticle based irrigants as an intracanal medicament. Calcium hydroxide and nano chitosan intracanal treatment showed a strong efficacy against*E. Faecalis* biofilm. This helps to lower the in periapcal infection and to improve the success of the endodontic procedure.

## Conclusion:

Both Nano Calcium Hydroxide and Chitosan nanoparticles showed considerable antibacterial activity against *Enterococcus faecalis* biofilms. The bactericidal potential of these nanoparticles was dependent on the maturity of the biofilms, with equal potential on 6-week-old biofilms and increased potential on more mature 10-week-old biofilms, with Nano Calcium Hydroxide showing better potential on mature biofilms. Further studies are required to enhance their potential on highly mature biofilms to completely eliminate biofilms.

## Figures and Tables

**Table 1:** Study groups based on intracanal medicament and biofilm age

**Main Group**	**Medicament used**	**Sub Group ( n = 7)**	**Age of Biofilm**
Group A	Nano-Calcium Hydroxide	A1	6 Week Old *E. Fecalis* Biofilm
		A2	10 Week Old *E. Fecalis* Biofilm
Group B	2% Chitosan Nano-Particle	B1	6 Week Old *E. Fecalis* Biofilm
		B2	10 Week Old *E. Fecalis* Biofilm

**Table 2:** *E. Faecalis* colony count (CFU ×10^-6^) in Nano Calcium Hydroxide and Chitosan nanoparticle groups at 6 and 10 weeks.

**Time intervals**	**Groups**	**N**	**Minimum**	**Maximum**	**Mean**	**Std.Deviation**
						
At 6 weeks	Nano Calcium	7	0	6.8	1.121	2.5146
	Hydroxide					
	Chitosan Nano	7	0	5.6	2.617	1.9028
	particle					
At 10	Nano Calcium	7	0	0.066	0.01	0.0246
weeks	Hydroxide					
	Chitosan Nano particle	7	0	3	0.948	1.0987
Independent t test;
*indicates a significant difference at p≤0.05

**Table 3:** Intergroup comparison of *E. Faecalis* colony count (CFU'sx10^-6^) and at 10 weeks

**Comparison groups**	**Time intervals**	**Mean Difference**	**t value**	**df**	**p value**
Nano Calcium Hydroxide vs Chitosan Nano	At 6 weeks	-1.4961429	-1.255	12	0.233
particle	At 10 weeks	-0.9385143	-2.259	12	0.043*
*p value <0.05 statistically significant,
<0.01 highly significant,
<0.001 very highly significant

**Table 4:** Intra group comparison of Faecalis colony count (CFU’sx10^-6^) among groups

**Intragroup comparison between different**		**Mean**	**t value**	**df**	**p value**
Time intervals		Difference			
Nano Calcium Hydroxide	At 6weeks vs	1.1109429	1.167	6	0.288
	At 10 weeks				
Chitosan nano particle	At 6weeks vs	1.6685714	1.718	6	0.137
	At 10 weeks				
*p value<0.05statisticallysignificant,
<0.01highlysignificant,
<0.001veryhighlysignificant
